# ﻿Rediscovering the dancing semislug genus *Cryptosemelus* Collinge, 1902 (Eupulmonata, Ariophantidae) from Thailand with description of two new species

**DOI:** 10.3897/zookeys.1076.75576

**Published:** 2021-12-08

**Authors:** Arthit Pholyotha, Chirasak Sutcharit, Somsak Panha

**Affiliations:** 1 Animal Systematics Research Unit, Department of Biology, Faculty of Science, Chulalongkorn University, Bangkok 10330, Thailand Chulalongkorn University Bangkok Thailand; 2 Academy of Science, The Royal Society of Thailand, Bangkok 10300, Thailand Academy of Science, The Royal Society of Thailand Bangkok Thailand

**Keywords:** Diversity, endemic, land snail, limestone, Malay Peninsula, systematics, taxonomy

## Abstract

Knowledge of Thai semislugs remains scarce, especially the dancing semislug genus *Cryptosemelus.* Prior to the present study, only a single species has been recognized with little available information. To address this knowledge gap, we surveyed for semislugs in western and southern Thailand, which yielded three species belonging to the genus *Cryptosemelus*. The little-known type species *C.gracilis* is redescribed herein, including a comparison with the type specimens. Two additional species, *C.betarmon***sp. nov.** and *C.tigrinus***sp. nov.**, are described as new to science. All three species are characterized by differences in their genital anatomy, especially with respect to anatomical details of the penis, epiphallus, and spermatophore. In addition, *C.tigrinus***sp. nov.** differs from *C.gracilis* and *C.betarmon***sp. nov.** in the mantle color pattern.

## ﻿Introduction

Becoming a slug through the reduction of the shell has occurred multiple times among the stylommatophoran land snails; this has occurred particularly frequently within the limacoid snail families Ariophantidae Godwin-Austen, 1883, Helicarionidae Bourguignat, 1877, and Urocyclidae Simroth, 1889 (Solem 1966; Hausdorf 1998; Hyman et al. 2007; Hyman and Köhler 2020). This process, so-called ‘limacization’, has also produced intermediate forms between slugs and snails, known as ‘semislugs’, which are characterized by the presence of an external shell with a reduced number of whorls, and a loss of the ability to completely withdraw its body under the protective shell (Collinge 1902; Blanford and Godwin-Austen 1908; Solem 1966; Barker 2001; Schileyko 2003; Schilthuizen and Liew 2008; Hyman and Köhler 2020). In addition, semislugs tend to show extensive development of the mantle lobes that can completely cover its shell, providing an increased surface area for gaseous exchange (Tillier 1983; Barker 2001). Located in the center of the Indo-Burma biodiversity hotspot (Myers et al. 2000), Thailand harbors a large number of snails, slugs, and semislugs belonging to the Ariophantidae and Helicarionidae (Solem 1966; Panha 1996; Hemmen and Hemmen 2001), of which several semislug genera have never been systematically revised since the seminal work of Solem (1966).

In the past decade, knowledge of the species diversity of Thailand’s ariophantid snails has dramatically improved, and many genera/species have been described and their systematics have been revised (i.e., Pholyotha et al. 2020, 2021). For the poorly known genus *Cryptosemelus* Collinge, 1902 such a systematic revision had been pending so far as there had been no additional records on this taxon since its first description. Collinge (1902) only gave a very brief description without providing any details on the genitalia, which bear important characters in semislug taxonomy (Blanford and Godwin-Austen 1908; Solem 1966; Hyman and Ponder 2010; Hyman and Köhler 2020). This monotypic genus was described based on specimens collected during the ‘The University of Cambridge Expedition to the North-Eastern Malay States and Upper Perak’, known as the ‘Skeat Expedition, 1899–1900’ (Gibson-Hill et al. 1953), which visited a region now situated in southern Thailand and the northern part of the Peninsular Malaysia. In addition, *Cryptosemelus* has been referred to as the ‘dancing semislug’ because of the fidgety or dance-like movement that it makes when it is disturbed or attacked (Collinge 1902).

The purely shell-based taxonomy of the semislug groups provides insufficient evidence for their systematic classification because the highly reduced and rather featureless shells provide a dearth of informative characters. Convergence in shell characters has been demonstrated in Australian helicarionids (Hyman and Köhler 2020). The lack of reproductive information has created a profound inaccuracy in their recognition and delimitation (e.g., Blanford and Godwin-Austen 1908; Solem 1966; Schilthuizen and Liew 2008; Hyman and Ponder 2010; Hyman and Köhler 2020). At present, anatomy-based approaches or those integrating molecular analyses are likely to more successfully resolve these taxonomic problems. Because the systematic revision of *Cryptosemelus* has never been undertaken for over a century, to fill this gap, we present here valuable data on the genus *Cryptosemelus*, especially the genitalia, spermatophore, mantle extensions, and radula. This paper includes a re-description of its type species, *C.gracilis* Collinge, 1902, and the descriptions of two additional new species.

## ﻿Materials and methods

**Figure 1.**
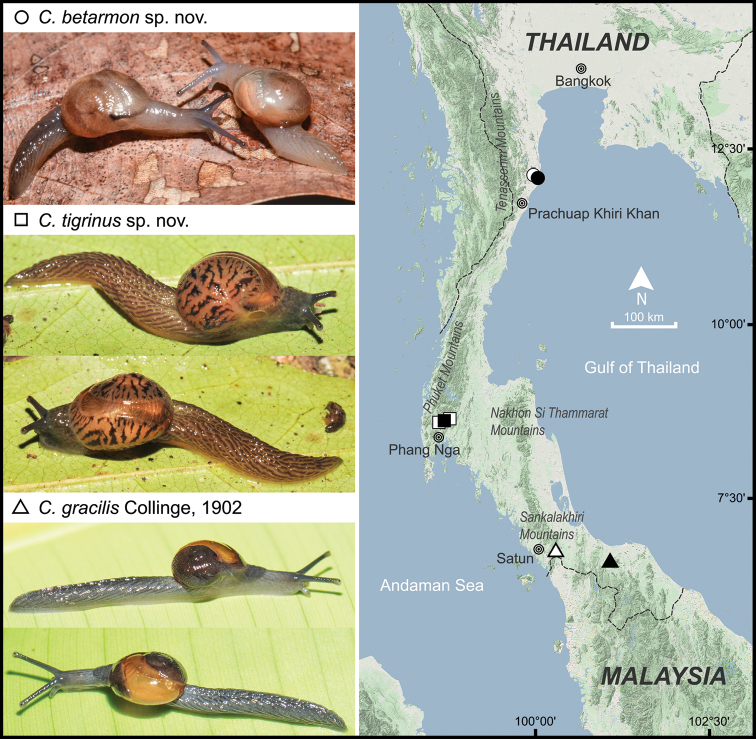
Geographic distribution and living animals of *Cryptosemelusgracilis*, *C.betarmon* sp. nov., and *C.tigrinus* sp. nov. based on the specimens examined herein. All not to scale. Black symbols indicate type locality and white symbols indicate other localities.

This study is based on voucher specimens deposited in the Chulalongkorn University Museum of Zoology, Bangkok, Thailand and new materials collected during the rainy season from western and southern Thailand (Fig. 1). Prior to preservation, collected semislug specimens were photographed in life. Each semislug was then euthanized following the standard two-step method protocols (American Veterinary Medical Association 2020), and then fixed in 95% (v/v) ethanol for morphological work. Species identification was made based on the literature, i.e., Collinge (1902), Blanford and Godwin-Austen (1908), and Solem (1966), and then compared with available type specimens and the reference collection of the University Museum of Zoology, Cambridge. For the descriptive work, adult shells were measured for size using a vernier caliper and counting the number of whorls. Three to ten specimens of each species were dissected and examined under an Olympus SZX2-TR30 stereoscopic light microscope. Shell and genitalia were imaged using a Nikon camera (DSLR D850) with a Nikon 105 Macro lens (AF-S VR Micro-Nikkor 105 mm f/2.8G IF-ED) and the inner sculpture of the genitalia was imaged using a stereo microscope with Cell’D Imaging Software. Radulae were extracted, soaked in 10% (w/v) sodium hydroxide, cleaned with distilled water, and then imaged by scanning electron microscopy (SEM; JEOL, JSM-6610 LV).

Descriptions of all new species herein are attributed to the first author. Type material and other voucher specimens are deposited in the Chulalongkorn University Museum of Zoology (**CUMZ**), Bangkok, Thailand and additional paratype specimens are deposited at the Natural History Museum, London, United Kingdom (**NHM** or **NHMUK**–when citing specimen lots deposited in the NHM) and the Zoological Reference Collection (**ZRC**) of the Lee Kong Chian Natural History Museum, National University of Singapore, Singapore.

The following abbreviations were used as defined by Blanford and Godwin-Austen (1908), Solem (1966), Pholyotha et al. (2020), and Sutcharit et al. (2020): **at**, atrium; **de**, diverticulum of epiphallus; **e1**, portion of epiphallus nearer to penis; **e2**, portion of epiphallus nearer to retractor muscle; **fo**, free oviduct; **gd**, gametolytic duct; **gs**, gametolytic sac; **hf**, head filament; **p**, penis; **pc**, penial caecum; **ldl**, left dorsal lobe; **lsl**, left shell lobe; **prm**, penial retractor muscle; **ps**, penial sheath; **pv**, penial verge; **rdl**, right dorsal lobe; **rsl**, right shell lobe; **ss**, sperm sac; **tf**, tail filament; **v**, vagina; and **vd**, vas deferens.

In the descriptions of the genitalia, the term ‘proximal’ refers to the region closest to the genital opening, while ‘distal’ refers to the region farthest away from the genital opening.

## ﻿Results

### ﻿Systematic description

#### Superfamily Helicarionoidea Bourguignat, 1877

**Family Ariophantidae Godwin-Austen, 1883**

#### Subfamily Ostracolethinae Simroth, 1901

##### ﻿Genus Cryptosemelus

Taxon classificationAnimaliaStylommatophoraAriophantidae

Collinge, 1902

49920EA0-6A64-5441-9259-8747E487586B

Cryptosemelus Collinge, 1902: 76. Blanford and Godwin-Austen 1908: 180. Thiele 1931: 640. Zilch 1959: 326. Vaught 1989: 97. Schileyko 2003: 1332. Bank 2017: 53. Inkhavilay et al. 2019: 75.

###### Type species.

*Cryptosemelusgracilis* Collinge, 1902, by monotypy.

###### Description.

Shell thin, subglobose to globose, and imperforate. Shell surface smooth, polished, and with pale yellowish to olive tinge or golden amber. Whorls 3½–4, rapidly increasing; body whorl large and rounded. Aperture oblique and crescentic with simple lip.

Animal with reticulated skin, pale grayish, brownish, blue-gray, and blackish body marked by conspicuous oblique lines running downwards and backwards. Mantle extensions well-developed and divided into two shell lobes and two dorsal lobes. Shell lobes entirely covering shell or retracted when disturbed; left and right shell lobes usually with same color as body and with or without irregular stripes; right shell lobe (rsl) broad and triangular; left shell lobe (lsl) narrow triangular and relatively small-sized. Right dorsal lobe (rdl) ovate to crescent-shaped and left dorsal lobe (ldl) undivided, larger, and crescent-shaped. Sole tripartite and lateral foot margin present. Caudal horn absent.

Genitalia with slightly short to moderately long penis, thin penial sheath, long to very long epiphallus, penial retractor muscle attached to epiphallus, and short to slightly long gametolytic duct. Epiphallic caecum, flagellum, and dart apparatus absent. Spermatophore with complex branching spines.

Radular teeth arranged in a wide U-shape with symmetrical tricuspid central tooth, asymmetrical tricuspid lateral teeth with square to oblong base-plate, and bicuspid marginal teeth with oblong plate.

###### Remarks.

Originally, Collinge (1902) referred this genus to the family Girasiidae, but later it was suggested to be placed under the subfamily Parmarioninae of the family Zonitidae (Blanford and Godwin-Austen 1908). Thiele (1931) then reclassified this genus, placing it under the subfamily Helicarioninae of the family Ariophantidae. This familial classification was then widely accepted and followed by subsequent authors except with the distinct subfamilial classification in which Zilch (1959) and Vaught (1989) placed *Cryptosemelus* as a member of the subfamily Macrochlamydinae, while Schileyko (2003) arranged it under the subfamily Parmarioninae. Regardless of the phylogenetic study, the higher classification of *Cryptosemelus* is still equivocal. Therefore, in this study, we follow the most recent gastropod classification that placed *Cryptosemelus* under the Ostracolethinae of the Ariophantidae (Bouchet et al. 2017).

**Figure 2.**
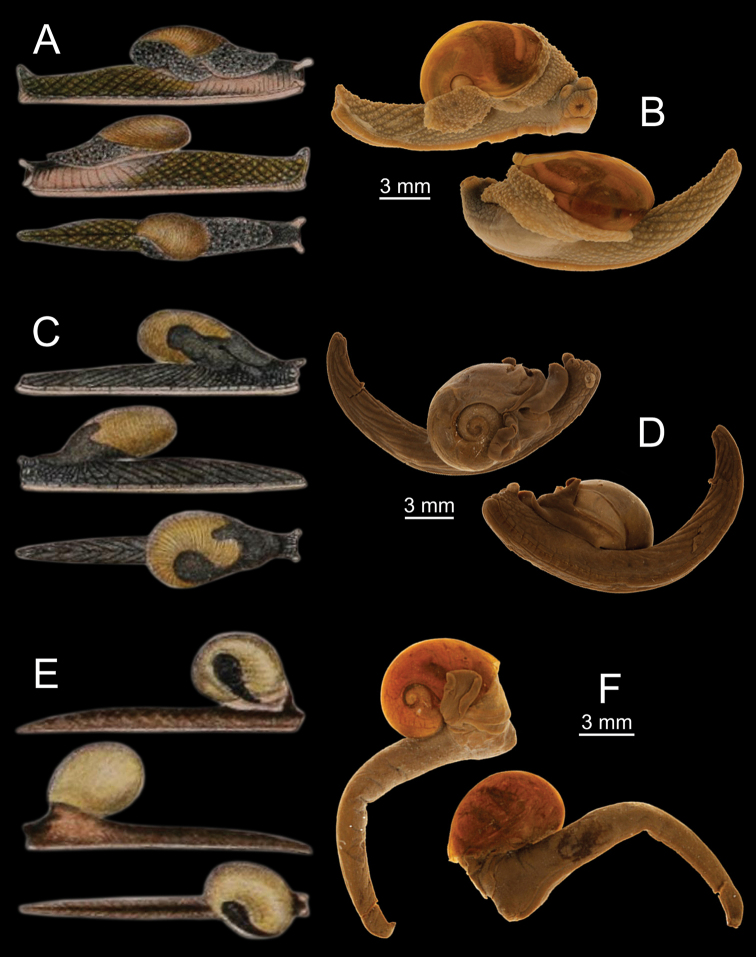
Comparison of shell and mantle lobes among the three monotypic semislugs described by Collinge (1902)**A, B***Apoparmarionpartridgii* Collinge, 1902 **A** original illustration and **B** syntype UMZC I.66414 **C, D***Cryptosemelusgracilis* Collinge, 1902 **C** original illustration and **D** syntype UMZC I.66448 **E, F***Paraparmarionelongatus* Collinge, 1902 **E** original illustration **F** syntype UMZC I.66522. Credits: **A, C, E** after Collinge (1902) and **B, D, F** online catalogues of the UMZC, Cambridge.

Collinge (1902) additionally described another two monotypic semislug genera, *Apoparmarion* and *Paraparmarion*, from Peninsular Malaysia based on specimens from the Skeat Expedition. These two genera differ from the genus *Cryptosemelus* mainly based on the number of shell whorls and mantle extensions, shape of the caudal horn, and genital structure. The genus *Apoparmarion* has a very reduced shell with about two whorls, with mantle extensions rising upon the shell on all sides with the right shell lobe posteriorly large, wing-like, and covering the apex of the shell, a prominent caudal horn, and genitalia with both a flagellum and dart apparatus (Fig. 2A, B; Collinge 1902). In contrast, *Cryptosemelus* has a reduced shell of about 3 to 4 whorls, with well-developed mantle extensions with the right shell lobe covering the apex and larger than the left shell lobe, a tail with no caudal horn, and genitalia without flagellum and dart apparatus. For further comparison, *Paraparmarion* and *Cryptosemelus* share a similar reduction in the number of shell whorls and the disappearance of the caudal horn, but *Paraparmarion* has only a right shell lobe (Fig. 2E, F; Collinge 1902), whereas *Cryptosemelus* has both right and left shell lobes (Fig. 2C, D; Collinge 1902). Unfortunately, the genitalia of the genus *Paraparmarion* have never been examined for comparison. A future search for additional specimens of the genus *Paraparmarion* is necessary for elucidating its relationship with the genus *Cryptosemelus*.

##### ﻿ Cryptosemelus gracilis

Taxon classificationAnimaliaStylommatophoraAriophantidae

Collinge, 1902

6A656FA5-21DB-5D36-AAC5-5A7F120FBA72

**Figure 3.**
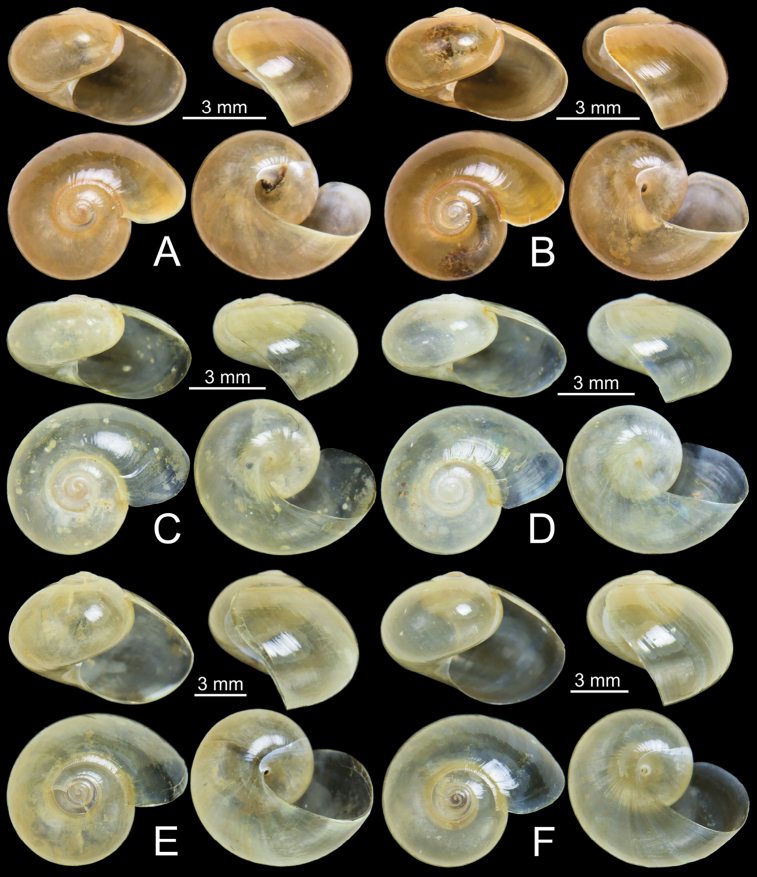
Shells **A, B***Cryptosemelusgracilis* specimen CUMZ 7954 **C, D***C.betarmon* sp. nov. **C** holotype CUMZ 7959, and **D** paratype CUMZ 7960 **E, F***C.tigrinus* sp. nov. **E** holotype CUMZ 7955, and **F** paratype CUMZ 7956.

**Figure 4.**
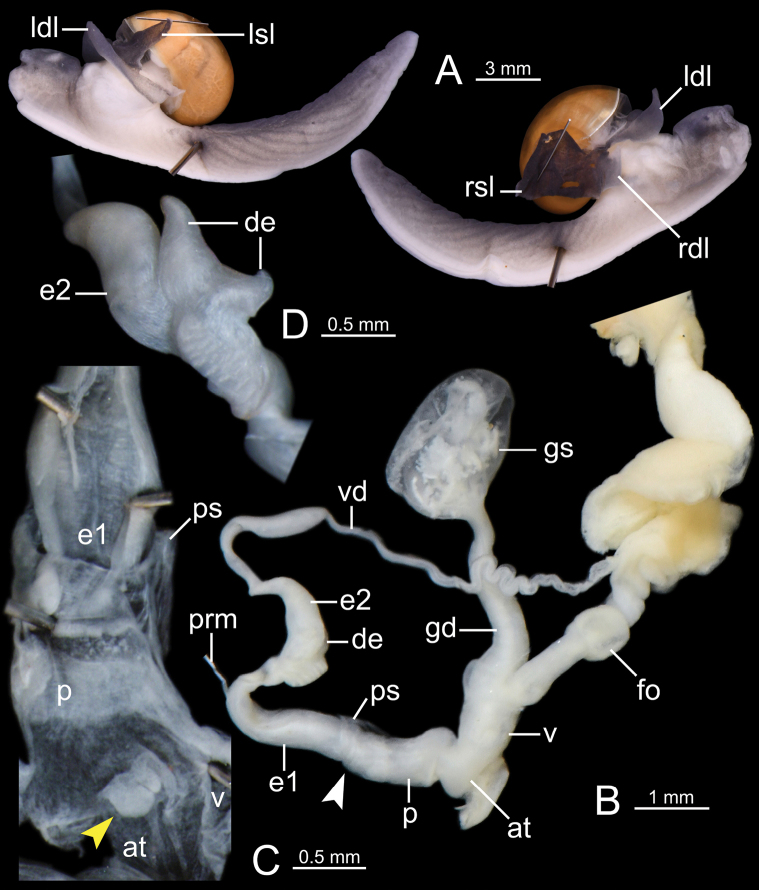
*Cryptosemelusgracilis* specimen CUMZ 7954 **A** both sides of animal showing four lobes of mantle and **B–D** genitalia: **B** general view of the genital system **C** internal structure of the penis, and **D** external structure of epiphallus (e2). White arrow indicates the end of the penis. Yellow arrow indicates the protruded tissue inside the penis near the atrium.

**Figure 5.**
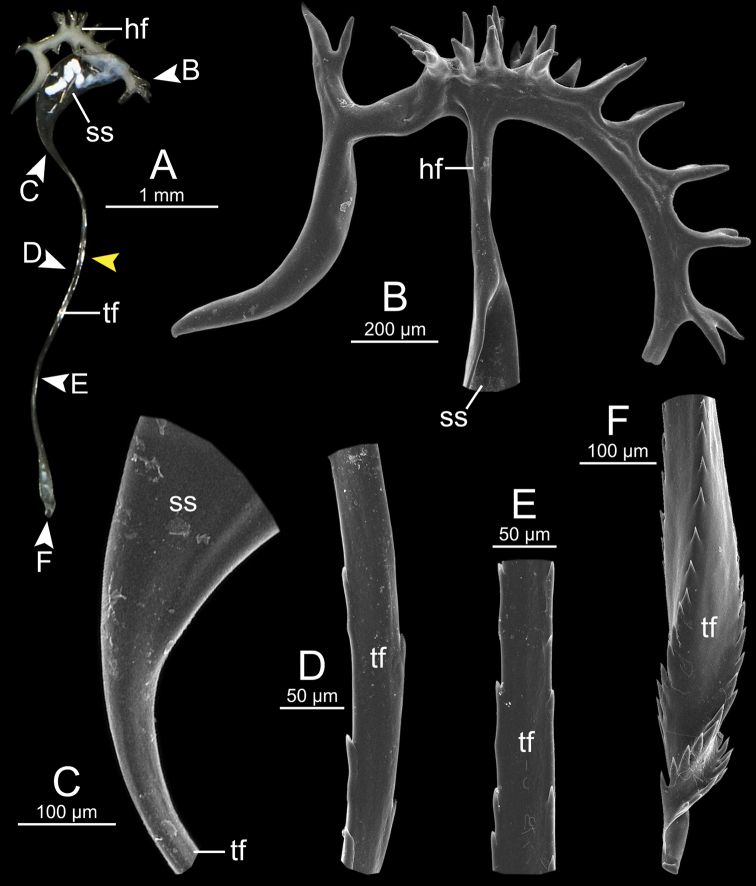
Spermatophore of *Cryptosemelusgracilis* specimen CUMZ 7954 **A** general view of the spermatophore **B–F**SEM images **B** head filament showing branching spines **C** region of tail filament near sperm sac showing no spine **D** alternate-spined region of tail filament near the end of spines from the tip **E** opposite-spined region of tail filament near the tip region, and **F** tail filament showing branching spines on the tip region. Yellow arrow indicates the end of spines from the tip.

Figs 1 , 2C, D , 3A, B , 4 , 5 , 10A

Cryptosemelus gracilis Collinge, 1902: 76, pl. 5, figs 37–39. Type locality: Bukit Besar, State of Nawng Chik [Nong Chik District, Pattani Province, Thailand]. Laidlaw 1933: 221. Benthem Jutting 1949: 71. Zilch 1959: 326. Maassen 2001: 112. Schileyko 2003: 1332.

###### Type material.

***Syntype*** UMZC I.66448 (one specimen in spirit; Fig. 2D) from Bukit Besar, Patani [Pattani Province, Thailand], Malay Peninsula.

###### Other material examined.

Ton Din, Khuan Don District, Satun Province, Thailand (6°43'N, 100°09'E): CUMZ 7954.

###### Diagnosis.

Shell globose and pale golden amber. Animal with blue-gray body. Genitalia with large vagina and elongated epiphallus with two small diverticula. Inner sculpture of penis with a small papilla near atrium. Spermatophore with a head filament of several spines and long tail filament with a cluster of small spines at the tip.

###### Description.

***Shell*** (Fig. 3A, B). Shell globose, small size (width up to 6.6 mm, height up to 4.2 mm), thin, smooth, polished, and pale golden amber. Whorls 3½–4, rapidly increasing; body whorl large and well-rounded at periphery. Spire slightly elevated; suture little impressed. Aperture oblique, diagonal, and roundly ovate; peristome simple and thin. Columellar margin simple. Umbilicus imperforate.

***Genital organs*** (Figs 4B–D, 5). Atrium (at) short. Penis (p) rather short, cylindrical, and with thin penial sheath covering entire penis. Internal penis surface nearly smooth, with small papilla (protruded tissue) near atrium (yellow arrow in Fig. 4C). Epiphallus (e1+e2) approximately three times total penis length; e1 cylindrical and gradually smaller in diameter (Fig. 4B); proximal e2 enlarged with irregularly undulated surface patch; and distal e2 generally smooth surface. Diverticulum (de) having two caeca: one small and one more muscular, thicker, and slightly larger (Fig. 4D). Penial retractor muscle (prm) thin and attached at junction between e1 and e2. Vas deferens (vd) thin tube connected between distal epiphallus and free oviduct (Fig. 4B).

Vagina (v) large, cylindrical, and approximately half of penis length. Gametolytic sac (gs) bulbous (Fig. 4B with spermatophore); gametolytic duct (gd) rather short, cylindrical, and somewhat broader at its base. Free oviduct (fo) cylindrical, approximately one and a half times penis length, and encircled with thick tissue in middle (Fig. 4B).

Spermatophore long (Fig. 5). Sperm sac (ss) enlarged and elongate ovalate. Head filament (hf) large and divided into two major branches located opposite: first branch bearing one small bifid spine, and second branch containing several bifid spines (Fig. 5B). Tail filament (tf) very long tube; terminal part about two-thirds of its length containing a series of tiny spines arranged in spiral rows (Fig. 5D–F).

***Radula*** (Fig. 10A). Teeth arranged in a wide U-shape with half row formula: 1–(19–20)–38 teeth. Central tooth square base-plate with symmetrical tricuspid; mesocone large and triangular shape; ectocones small and pointed cusps. Lateral teeth asymmetrical tricuspid, inner teeth square base-plate and then gradually become elongate-shaped at outer teeth. Inner lateral teeth with mesocone large, triangular, and with pointed cusp; ectocone larger than endocone and located near tooth base. Outer lateral teeth: mesocone and ectocone large and pointed tip; endocone very small to nearly absent. Marginal teeth starting at approximately teeth numbers 19 to 20 with obliquely elongate bicuspid; endocone large and pointed tip; ectocone small lanceolate shape with pointed cusp. Outermost marginal teeth shorter and smaller than inner teeth.

***External appearance*** (Figs 1, 2C, D, 4A). Living animal with reticulated skin, blue-gray to blackish body marked by conspicuous oblique grooves running downwards. Four mantle extensions well-developed and same color as body. Shell lobes enlarged to cover entire shell; left shell lobe (lsl) smaller than right shell lobe (rsl); left dorsal lobe (ldl) larger than right dorsal lobe (rdl). Sole divided into three parts longitudinally. Caudal horn absent.

###### Distribution, habitat, and behavior observations.

*Cryptosemelusgracilis* can be found in Satun, Yala, Songkhla, and Pattani Provinces in southern Thailand (Fig. 1). We searched after rain and found the semislug populations normally hiding under the slope of rocks or the tree trunks, and sometimes climbing on the rocks or low branches of plants. When the semislug are disturbed, they escape by quickly flipping and wagging their tail, and then falling on the floor. Information on its natural predators and parasites remains scarce, but the carnivorous slug genus *Atopos* and streptaxid snails were found sympatrically with this semislug.

###### Remarks.

A specimen of *C.gracilis* was first discovered from ‘Bukit Besar’, the Malay Language, which means ‘Big Mountains’ in Thai Language (Skeat 1901; Annandale and Robinson 1906). However, this type locality is now referred to as the Namtok Sai Khao National Park area that is situated on the boundary of Pattani, Yala, and Songkhla Provinces in southern Thailand.

In this study, we examined specimens from Satun Province, which are identical to the syntype in having a blue-gray body with prominent oblique lines running downwards on the posterior body, large right shell lobes that covered the apex of the shell, and no caudal horn. Benthem Jutting (1949) provisionally attributed three semislug specimens from Telom Valley, Gunong Siku, Pahang State (1,000 m altitude) as *C.gracilis* s.l., but this was without any description or illustration. Based on our observation, all recognized *Cryptosemelus* species generally have a restricted distribution, and tend to occur at low altitudes near the mean sea level. Therefore, we consider that those semislug specimens from Pahang State probably belong to a distinct taxon from *C.gracilis* s.s. However, this semislug population needs to be re-examined to confirm their taxonomic status.

##### ﻿ Cryptosemelus betarmon

Taxon classificationAnimaliaStylommatophoraAriophantidae

Pholyotha sp. nov.

C45E9575-604B-54F3-BE16-5DF26B55FF83

http://zoobank.org/11AF3310-99EB-402D-8A3C-68AD48B349DE

**Figure 6.**
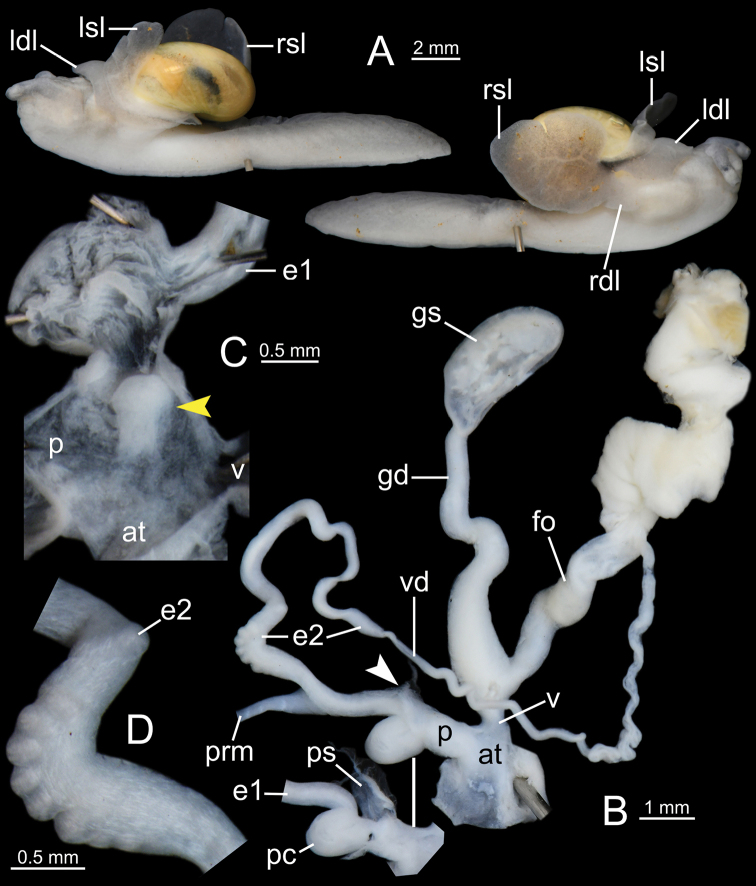
*Cryptosemelusbetarmon* sp. nov. paratype CUMZ 7960 **A** both sides of animal showing four lobes of mantle **B–D** genitalia **B** general view of the genital system **C** internal structure of the penis, and **D** external structure of epiphallus (e2). White arrow indicates the end of the penis. Yellow arrow indicates the protruded tissue inside the penis near the atrium.

**Figure 7.**
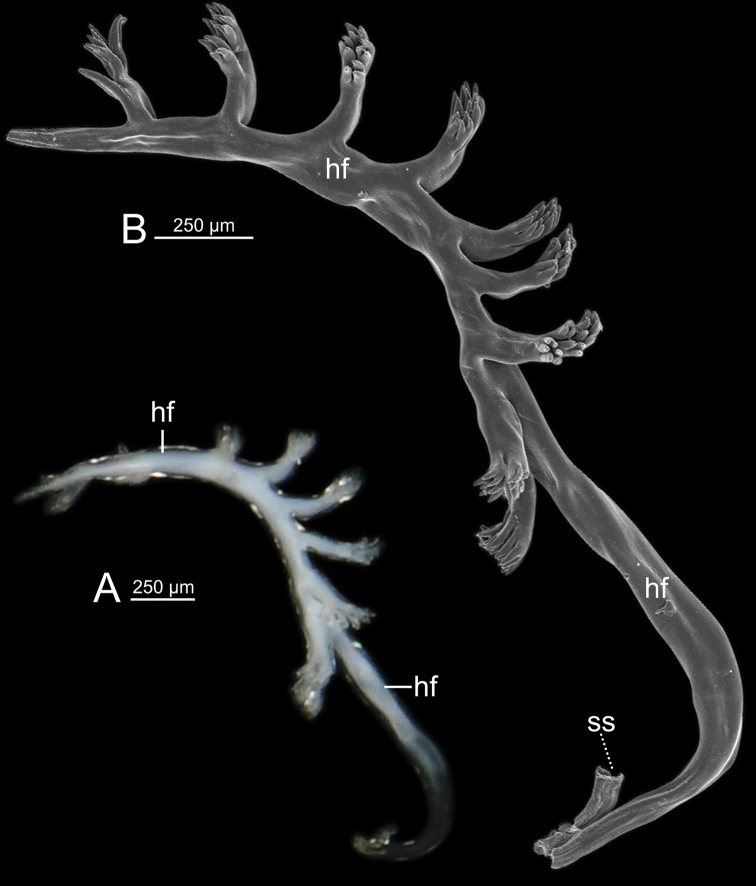
Spermatophore of *Cryptosemelusbetarmon* sp. nov. paratype CUMZ 7960 **A** General view of head filament and **B**SEM images of head filament showing branching spines.

Figs 1 , 3C, D , 6 , 7 , 10B

###### Type material.

***Holotype*.**CUMZ 7959 (Fig. 3C, width 7.4 mm, height 4.1 mm).

***Paratypes*.** Same locality as holotype: CUMZ 7960 (Fig. 3D, width 7.3 mm, height 4.1 mm), NHMUK (two shells), and ZRC (two shells). Limestone outcrops at Sam Roi Yot District, Prachuap Khiri Khan Province, Thailand (12°14'N, 99°55'E): CUMZ 7961.

###### Type locality.

Limestone outcrop at Wat Bang Pu, Sam Roi Yot District, Prachuap Khiri Khan Province, Thailand (12°12'N, 100°00'E).

###### Diagnosis.

Shell depressedly subglobose and pale yellowish. Animal with grayish body. Genitalia with penial caecum, small vagina, and elongated epiphallus. Inner sculpture of penis with papilla and penial caecum. Spermatophore with a row of branching spines.

###### Description.

***Shell*** (Fig. 3C, D). Shell depressedly subglobose, small size (width up to 7.4 mm, height up to 4.1 mm), thin, smooth, polished, pale yellowish with olive tinge. Whorls 3½–4, rapidly increasing; body whorl large and well-rounded at periphery. Spire slightly elevated; suture little impressed. Aperture oblique, diagonal, roundly ovate; peristome thin, simple. Columellar margin simple. Umbilicus imperforate.

***Genital organs*** (Figs 6B–D, 7). Atrium (at) short. Penis (p) somewhat short, cylindrical, and with thin penial sheath (ps) covering most of the penis; penial caecum (pc) enlarged and bulbous. Internal sculpture of proximal penis covered by nearly smooth surface and with thickened papilla (protruded tissue); and in penial caecum covered by irregularly folds (Fig. 6C). Epiphallus (e1+e2) approximately four times total penis length; e1 shorter than e2 and cylindrical; proximal e2 enlarged and with undulated surface (Fig. 6D) and then gradually reduced diameter to distal end (Fig. 6B). Penial retractor muscle (prm) thick, enlarged at base and attached at junction between e1 and e2. Vas deferens (vd) thin tube connected between distal epiphallus and free oviduct (Fig. 6B).

Vagina (v) cylindrical, and slightly shorter than a half of penis length. Gametolytic sac (gs) bulbous (Fig. 6B with spermatophore); gametolytic duct (gd) cylindrical, enlarged at base, and then gradually reduced in diameter to gametolytic sac. Free oviduct (fo) long, cylindrical, approximately two times total penis length, and encircled with thick tissue in middle (Fig. 6B).

Spermatophore incomplete (sperm sac and tail filament missing). Head filament (hf) with nine branching spines arranged in a single row along the head filament section (Fig. 7).

***Radula*** (Fig. 10B). Teeth arrangement and shape similar to those of *C.gracilis*. Teeth with half row formula: 1–(27–28)–37 teeth. Central tooth square base-plate with symmetrical tricuspid. Inner lateral teeth square base-plate with asymmetrical tricuspid; outer lateral teeth oblong to elongate tricuspid. Marginal teeth elongate bicuspid. Marginal teeth starting at about teeth numbers 27–28; outermost teeth shorter and smaller than inner teeth.

***External appearance*** (Figs 1, 6A). Living animal with reticulated skin, pale to dark grayish body marked by prominent, oblique, pale brownish lines running downwards. Four mantle extensions well-developed and same color as body. Shell lobes enlarged to cover almost entire shell; right shell lobe (rsl) larger than left shell lobe (lsl); right dorsal lobe (rdl) smaller than left dorsal lobe (ldl). Foot sole divided into median and lateral planes. Caudal horn absent.

###### Etymology.

The specific name “*betarmon*” is from the Greek word meaning a dancer and refers to the fidgety movements or dance-like movements of living semislugs found in the field after being disturbed.

###### Distribution, habitat, and behavior observations.

*Cryptosemelusbetarmon* sp. nov. is restricted to the limestone outcrops in Prachuap Khiri Khan Province, Thailand (Fig. 1). During the rainy season, but with low precipitation, the semislugs were found inactive under the decaying leaf litter or sometimes inside the hole of decaying wood. This semislug species also moved quickly as well as quickly flipping and wagging its tail to escape after being disturbed. The data on its natural enemies are unknown, but the carnivorous snail, *Haploptychius* sp. (Streptaxidae), was found at a high abundance in the type locality.

###### Remarks.

This new species is a small-sized *Cryptosemelus* species which has a subglobose and pale yellowish shell with an olive tinge, and genitalia with a penial caecum and without an epiphallic diverticulum. Compared to the type species, this species has a globose and pale golden amber shell, genitalia with two small diverticula on the epiphallus, and no penial caecum.

##### ﻿ Cryptosemelus tigrinus

Taxon classificationAnimaliaStylommatophoraAriophantidae

Pholyotha sp. nov.

3E844FCD-BFA8-5A07-818F-B8BB626947DF

http://zoobank.org/98028C74-C2C5-4464-AE95-23FD93F25846

**Figure 8.**
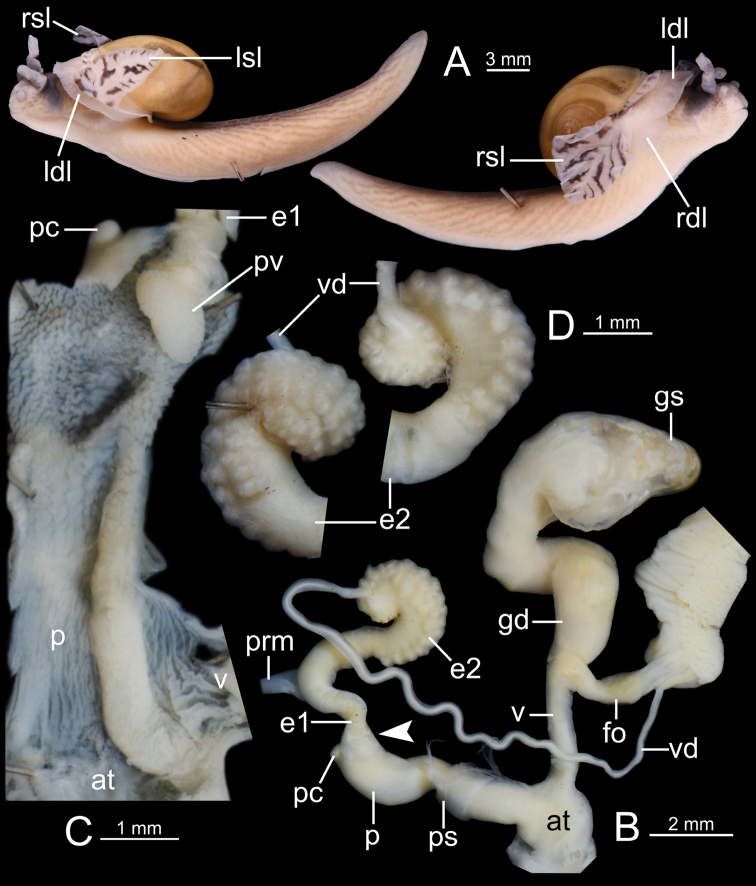
*Cryptosemelustigrinus* sp. nov. paratype CUMZ 7956 **A** both sides of animal showing four lobes of mantle **B–D** genitalia **B** general view of the genital system **C** internal structure of the penis, and **D** external structure of epiphallus (e2). White arrow indicates the end of the penis.

**Figure 9.**
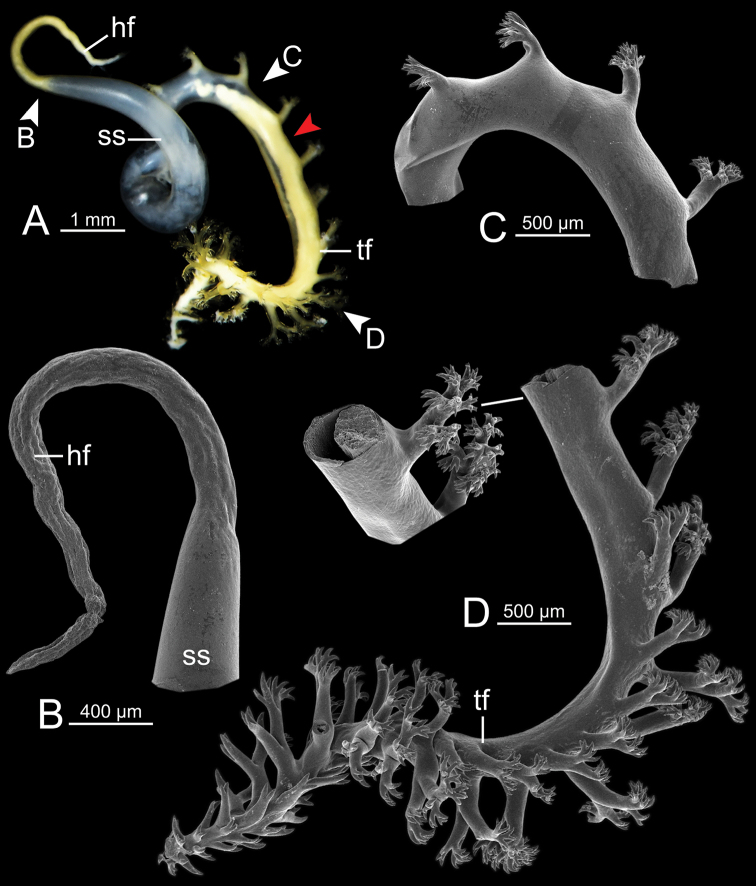
Spermatophore of *Cryptosemelustigrinus* sp. nov. paratype CUMZ 7956 **A** general view of the spermatophore **B–D**SEM images **B** head filament without spine **C** branching spines on the unclear boundary between sperm sac and tail filament, and **D** tail filament showing branching spines; inset showing cross section of tail filament. Red arrow indicates position of cross section of tail filament.

**Figure 10.**
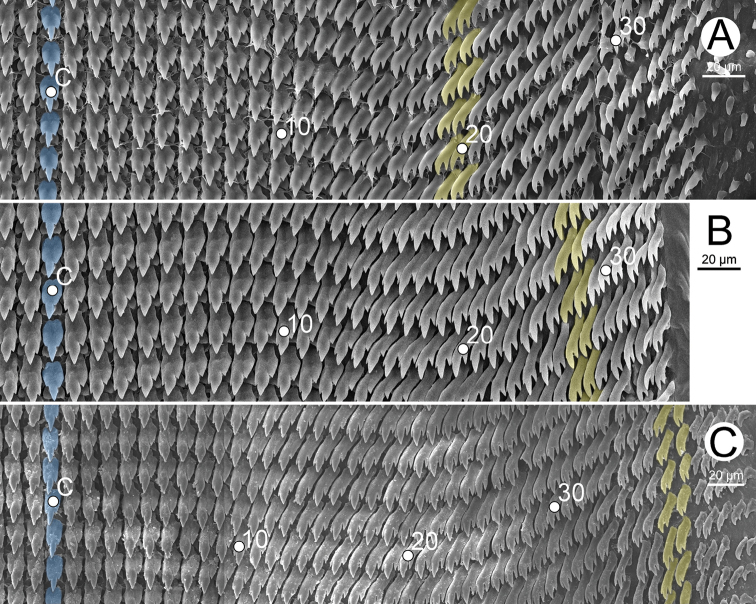
Representative SEM images of the radula **A***Cryptosemelusgracilis* specimen CUMZ 7954 **B***C.betarmon* sp. nov. paratype CUMZ 7960, and **C***C.tigrinus* sp. nov. paratype CUMZ 7956. Central tooth indicated by ‘C’; blue color indicates central tooth row; yellow color indicates the transition of outer lateral to marginal teeth.

Figs 1 , 3E, F , 8 , 9 , 10C

###### Type material.

***Holotype*.**CUMZ 7955 (Fig. 3E; width 10.7 mm, height 7.6 mm). ***Paratypes*.** Same locality as holotype: CUMZ 7956 (Fig. 3F; width 9.8 mm, height 6.8 mm), NHMUK (two shells), and ZRC (two shells). Limestone outcrops at Wat Suwan Khuha, Takua Thung District, Phang-Nga Province, Thailand (8°25'N, 98°28'E): CUMZ 7957. Limestone outcrops at Wat Tham Bang Toei, Mueang District, Phang-Nga Province, Thailand (8°27'N, 98°34'E): CUMZ 7958.

###### Type locality.

Limestone outcrop at Tham Phung Chang, Mueang District, Phang-Nga Province, Thailand (8°26'N, 98°30'E).

###### Diagnosis.

Shell globose, pale yellowish. Animal with brownish body, shell lobes pale yellowish-orange and flanked with irregular black bands. Genitalia with long penis and vagina and epiphallus with granulated surface near vas deferens; penial caecum and penial verge present. Inner sculpture of penis: proximal part with one thickened longitudinal fold; distal part with irregular folds. Spermatophore with smooth head filament and long tail filament with several delicate, branching spines.

###### Description.

***Shell*** (Fig. 3E, F). Shell globose, medium-sized (width up to 10.7 mm, height up to 7.6 mm), thin, smooth, polished, pale yellowish with an olive tinge. Whorls 4–4½, rapidly increasing; last whorl large and rounded at periphery. Spire elevated; suture little impressed. Aperture oblique, diagonal, roundly ovate; peristome thin, simple. Columellar margin simple. Umbilicus imperforate.

***Genital organs*** (Figs 8B–D, 9). Atrium (at) short. Penis (p) moderately long, cylindrical with thin penial sheath (ps) covering nearly half of its length; penial caecum (pc) small. Internal wall of penis: proximal part covered with very thin longitudinal folds and one thickened longitudinal fold; distal part with irregularly zigzag folds surrounding the penial verge. Penial verge (pv) elongate ovate shape and smooth surface (Fig. 8C). Epiphallus (e1+e2) equal to penis length; e1 slightly shorter than e2 and cylindrical; proximal e2 cylindrical and smooth surface (Fig. 8B); distal e2 cylindrical with prominently granulated surface (Fig. 8D). Penial retractor muscle (prm) thick, enlarged at base and attached at junction between e1 and e2. Vas deferens (vd) thin tube connected between distal epiphallus and free oviduct (Fig. 8B).

Vagina (v) long, slender, and approximately half of penis length. Gametolytic sac (gs) bulbous (Fig. 8B with spermatophore); gametolytic duct (gd) somewhat enlarged and cylindrical. Free oviduct (fo) cylindrical, about half of penis length, and encircled with thick tissue in middle (Fig. 8B).

Spermatophore long and twisted cylindrical tube (Fig. 9). Head filament (hf) elongate tube with smooth surface (Fig. 9B). Sperm sac (ss) enlarged, elongate ovate with unclear boundary between sperm sac and tail filament. Tail filament (tf) very long and enlarged tube with series of long and delicate branching spines arranged in a row, and then near the tip having multiple rows of short branching spines (Fig. 9C, D).

***Radula*** (Fig. 10C). Teeth arrangement and shape similar to those of *C.gracilis*. Teeth with half row formula: 1–(38–39)–44 teeth. Central tooth square base-plate with symmetrical tricuspid. Inner lateral teeth square base-plate with asymmetrical tricuspid; outer lateral teeth with oblong to elongate teeth with tricuspid. Marginal teeth elongate bicuspid. Marginal teeth starting at about teeth numbers 38–39; outermost teeth shorter and smaller than inner teeth.

***External appearance*** (Figs 1, 8A). Living animal with reticulated skin, pale to dark brownish body marked with prominent, oblique, dark brownish lines running downwards. Mantle extensions well-developed. Shell lobes pale yellowish-orange, painted with irregular black stripes, and enlarged to cover entire shell; right shell lobe (rsl) large (square shape in preserved specimen); left shell lobe (lsl) small (triangular shape in preserved specimen); left dorsal lobe (ldl) larger than right dorsal lobe (rdl). Foot sole divided into median and lateral planes. Caudal horn absent.

###### Etymology.

The specific name is a Latin word “*tigrinus*”, a noun in apposition referring to the dark stripes on shell lobes, which is similar to the color pattern of the tiger.

###### Distribution, habitat, and behavior observations.

*Cryptosemelustigrinus* sp. nov. can be found on the limestone hills in Phang-Nga Province (Fig. 1). This new semislug species has a high activity level, and is abundant in moist weather conditions after rain. They were seen hanging, crawling, or slowly climbing on the wet surface of the limestone rocks, tree trunks, and limestone shrubs. This new species also has an escape behavior similar to the other congeners. Its predators are unknown, but the carnivorous slug *Atopos* sp. (Rathuisiidae) and *Discartemon* sp. (Streptaxidae) were sympatric with the new species.

###### Remarks.

*Cryptosemelustigrinus* sp. nov. differs from *C.gracilis* and *C.betarmon* sp. nov. in having pale yellow-orange banded shell lobes and a well-developed penial verge, whereas *C.gracilis* and *C.betarmon* sp. nov. have monochrome shell lobes and do not have a penial verge.

## ﻿Discussion

The three character states of (i) reduced shell, (ii) presence of the stimulator with a calcareous dart, and (iii) attachment of the penial retractor muscle directly to the epiphallus rather than to the epiphallic caecum are characteristic for members of the Ostracolethinae, family Ariophantidae (Hausdorf 1998). This study contains the first anatomical investigation of *Cryptosemelus* and we found that its genital anatomy is consistent with a membership in this subfamily. This finding is corroborated by evidence from shell and reproductive characters as outlined above (dart possibly secondarily reduced). With regard to the absence of a dart apparatus, this character together with the epiphallic caecum and flagellum appears to have been lost and gained repeatedly and convergently among the Ariophantidae and the limacoid snails in general (Hausdorf 1998; Schileyko and Semenyuk 2018).

The shell morphology of the genus *Cryptosemelus* is similar to that of several semislug genera on mainland Southeast Asia that consist of *Apoparmarion*, *Cryptaustenia* Cockerell, 1891, *Durgella* Blanford, 1863, and *Paraparmarion*. However, the absence of a caudal horn, which is a unique character shared between *Cryptosemelus* and *Paraparmarion*, distinguishes these two semislug genera from the others (Collinge 1902; Blanford and Godwin-Austen 1908; Solem 1966; Schileyko 2002, 2003).

*Cryptosemelus* was stated to differ from *Paraparmarion* in that the left shell lobe is well-developed, whereas it is missing in the latter (Collinge 1902). However, the presence and absence of the left shell lobe can simultaneously occur within congeneric species in the ariophantid snail genus *Sarika* Godwin-Austen, 1907 (Pholyotha et al. 2020). Hence, the relationship between *Cryptosemelus* and *Paraparmarion* remains uncertain since they were consecutively described in the same publication without genital information (Collinge 1902). However, the genitalia of *Cryptosemelus* have been examined herein and its generic status is confirmed. The genital morphology of all species of *Cryptosemelus* examined herein all show no epiphallic caecum, flagellum, and dart apparatus. In comparison, a flagellum occurs only in *Apoparmarion*, while an epiphallic caecum occurs only in *Durgella* (Collinge 1902; Blanford and Godwin-Austen 1908; Solem 1966; Schileyko 2002, 2003).

Regarding the dart apparatus, the main role of which is for stimulation during courtship behaviour, this character has been used as a distinguishing character among the limacoid genera, i.e., *Hemiplecta* Albers, 1850 (with dart apparatus) vs. *Falsiplecta* Schileyko & Semenyuk, 2018 (without dart apparatus), or *Macrochlamys* Gray, 1847 (with dart apparatus) vs. *Syama* Godwin-Austen, 1908 (without dart apparatus). These sibling genera have a similar external appearance except for the dart apparatus (Blanford and Godwin-Austen 1908; Schileyko 2002, 2003; Schileyko and Semenyuk 2018). However, it is widely accepted that the dart apparatus could be present or absent within the same genus, i.e., *Cryptaustenia* and *Durgella* (Blanford and Godwin-Austen 1908; Solem 1966; Schileyko 2002, 2003). Moreover, no molecular phylogeny has been implemented to test the monophyly of the genera *Cryptaustenia* and *Durgella*.

In this study, the shell lobes and genitalia (penis and epiphallus) are considered as taxonomically informative and these can be used to distinguish all *Cryptosemelus* species. In addition, these characters might reflect the relationships among three species of *Cryptosemelus.* Our results indicated that *C.gracilis* is closely related to *C.betarmon* sp. nov. even though the distribution of *C.gracilis* is closer to *C.tigrinus* sp. nov. (Fig. 1). In support, the shell lobes of *C.gracilis* and *C.betarmon* sp. nov. have a monotone color and the internal wall of the penis has a papilla located close to atrium, whereas *C.tigrinus* sp. nov. has shell lobes with dark stripes and a large, conical penial verge. In addition, the undulated surface on the epiphallus of the two species is located close to the penial retractor muscle, while in *C.tigrinus* sp. nov. this character is located near the vas deferens. Furthermore, the color pattern of the shell lobes and the reproductive tracts of *C.tigrinus* sp. nov. are very distinct and unique, which could possibly be recognized as different subgenera or genera. However, we refrain from nominating this because at present it is without support from a molecular framework. Thus, future studies need more materials from other members of *Cryptosemelus* and will also require combining with molecular phylogenetic analyses to investigate this hypothesis.

Predator-prey interactions are recognized as major processes promoting morphological and behavioral diversity (Vermeij 1987; Chiba 2007; Morii et al. 2016; Sugiura 2016; Le Ferrand and Morii 2020). Land snails are preyed upon by a wide range of predators (e.g., rodents, birds, snakes, insects, and molluscs). Their own anti-predator adaptations, a passive defense by pulling their body into their shell and an active behavior by swinging their shell around, are well documented and are potentially associated with differences in shell traits (Vermeij 1987, 1993; Chiba 2007; Baalbergen 2014; Liew and Schilthuizen 2014; Morii et al. 2016; Morii and Wakabayashi 2017; Le Ferrand and Morii 2020). In contrast, the evolution of slug and semislug forms have sacrificed the protection offered by possession of a shell for the mobility, fast body movements, and ability to occupy very small spaces afforded by the reduction or elimination of the shell (Solem 1974; Barker 2001; Wiktor 2002; Dedov et al. 2019). In our study, this dancing semislug genus showed an anti-predator behavior by quick dance-like movements or the sudden flipping and wagging tail movement and the fast movement away from the location of threat. This behavior has also been recorded in several helicarionid semislugs: *Cryptaustenia* species in Papua-New Guinea, *Laocaia* species in Vietnam, and *Muangnua* species in Thailand (Wiktor 2002; Dedov et al. 2019; Tumpeesuwan and Tumpeesuwan 2019).

## Supplementary Material

XML Treatment for Cryptosemelus

XML Treatment for Cryptosemelus gracilis

XML Treatment for Cryptosemelus betarmon

XML Treatment for Cryptosemelus tigrinus
